# Corruption perceptions in the Schengen Zone and their relation to education, economic performance, and governance

**DOI:** 10.1371/journal.pone.0301424

**Published:** 2024-07-24

**Authors:** Ionela Munteanu, Bogdan-Vasile Ileanu, Iulia Oana Florea, Kamer-Ainur Aivaz

**Affiliations:** 1 Faculty of Economic Studies, Department of Finance and Accounting, Ovidius University of Constanta, Constanta, Romania; 2 Center for Health Outcomes and Evaluation, Bucharest, Romania; 3 Department of Finance and Accounting, Bucharest University of Economic Studies, Bucharest, Romania; 4 Faculty of Economic Studies, Department of General Economy, Ovidius University of Constanta, Constanta, Romania; Cavendish University / Kyambogo University, UGANDA

## Abstract

In the quest for integrity and transparency, the perception of corruption within a state not only undermines trust in governance but also hinders sustainable progress. This study investigates the relationship between education, economic performance, and governance and their impact on the assessment of corruption in the context of sustainable development goals. The research framework included data from 14 European countries, members of the Schengen zone, using panel data for the period 2003–2022. The analysis of the sample indicators guided us to apply further advanced research methods. Principal Components Analysis was applied to reduce dimensionality. Under various assumptions related to heterogeneity, stationarity and exogeneity, panel Fixed Effects (FE) and Dynamic Panel with Generalized Method of Moments (GMM) were applied to link the drivers and the perception of corruption. The results are twofold: (i) on the one hand, the diachronic evolution of corruption, education index, economic performance or governance perceptions reveals different patterns for the countries of the Schengen zone; (ii) on the other hand, the composite index for education and governance assessment had a significant impact on corruption perceptions, while economic performance showed no apparent link to corruption. Incorporating the effects of economic and political shocks, the study will explore the resilience of the Schengen Zone’s governance against corruption, particularly by examining the effectiveness of crisis management and education’s role in reinforcing integrity. It will also conduct a longitudinal analysis to assess how these shocks influence corruption perceptions over time, offering insights into the adaptability of policy responses and their lasting impact on public trust. The findings establish new research pathways for academics and provide valuable incentives to policymakers and professional trainers to improve mechanisms for combating corruption.

## 1.Introduction

General concern about the implementation of effective policies to achieve sustainable development goals has led to new research directions examining various factors that may influence these outcomes [1]. Among these, corruption perception plays a pivotal role, affecting both the trust in governance and the efficiency of economic systems. This study aims to delve into the comparative analysis of corruption perceptions within the Schengen Zone, investigating the impact of education, economic performance, and governance.

While sustainable economic development is often associated with industrialization and technological progress, it is also closely tied to governance quality and the robustness of educational systems [2]. These elements are significant for they can both foster and hinder sustainable development depending on their nature and implementation. In this regard, government institutions, economic strategists, and academic researchers have sought to understand and mitigate the adverse impact of corruption on economic value creation [3].

The literature suggests that the causes of corruption are complex and multifaceted, often influenced by economic disparity, varying levels of education, and the effectiveness of governance [4,5]. The perception of corruption varies significantly across countries with different income levels and between public and private sectors. It is also influenced by the presence or absence of internal control systems, such as internal audits, risk assessments, and mitigation procedures [6]. These systems can shape the risk perceptions of corruption within organizations [7,8].

This study shifts the focus to the Schengen zone countries, where corruption is not the norm but rather exceptions in a collective effort to uphold strong governance and policy coherence. The research question guiding this paper is: what is the role of education, economic performance, and perceived governance efficiency in assessing corruption in the context of sustainable development goals.

This paper contributes to the literature on corruption by proposing a novel approach that integrates quantitative economic data with qualitative data, derived from composite indicators of education and governance efficiency that reflect local public perception across various Schengen countries. Furthermore, it employs a mixed-method theoretical framework, incorporating theories such as the bad-apple theory, correlation theory, and organizational culture theory, to examine the nuanced public perception of fraud and corruption [9].

Moreover, the historical and cultural backdrop of Schengen countries imparts distinct attitudes towards corruption. The interplay between long-standing norms and the contemporary push for ethical standards creates a complex mosaic of public opinion. This is further complicated by the diversity of legal frameworks across the region, where the rigidity and enforcement of anti-corruption laws vary, influencing both the incidence and perception of corrupt activities.

The importance of civil society and public involvement is clear and significant. When people actively participate in society, especially when educated to understand and uphold civic duties, it effectively helps prevent corruption. The media also plays a critical role; when it operates without restrictions and with a commitment to ethical standards, it significantly influences how corruption is viewed and understood by the public.

Cooperation among Schengen Zone countries yields ambivalent outcomes. On one hand, it promotes the synchronization of anti-corruption policies and joint efforts to combat corrupt activities. On the other hand, it potentially facilitates transnational corrupt practices that can bypass the regulatory powers of individual states. As a result, the issue of corruption goes beyond the boundaries of individual countries and necessitates analysis within an international framework, especially where various national policies and economic engagements intersect.

The governance models of Schengen countries offer a spectrum of approaches to public administration, from centralized to decentralized systems, each with its nuances in discretion and potential for corrupt practices. Here, economic inequality emerges as a critical factor. Imbalances in the distribution of wealth and disparities in access to economic opportunities could create an environment where corrupt activities are more likely to occur. Alternatively, effective remediation of these disparities can facilitate the development of a society characterized by greater equity and justice.

The different levels of economic development across Schengen countries provide a solid foundation for analysis. Economic characteristics such as the degree of industrialization and the advancement of the service sector are critical in determining the level of corruption. These fundamental economic factors significantly influence how the public views and expects their governance systems to perform and uphold integrity.

Finally, the effectiveness of existing policy measures and the imperative for reforms are crucial elements for consideration. Public attitudes towards anti-corruption initiatives greatly affect their trust in governmental institutions. As we extend our analysis to the Schengen area, we aim to incorporate a wider range of factors, including those related to education, economic performance, and governance. By weaving these factors into our current analytical model, we expect to enhance our comprehension and provide a more detailed exploration of the diverse elements that contribute to public perceptions of corruption. These perceptions are shaped by a comprehensive array of influences, spanning from educational and economic influences on the effectiveness of governance systems.

Many authors, among them Aiston and Mukhametova [10], Lee et al. [11], Mangafic and Veselinovic [12] or Linhartová and Halásková [13] placed the assessment of corruption as a result variable, depending on the interconnection between a set of economic, socio-cultural, and political determinants pointing to different levels of corruption determination. Various determinants like demographic, religion, age, socio-cultural, economics or political components are found to be statistically significant in different types of models. Apparently, there is no study to confirm the contribution of all factors in the same model. Moreover, it seems that only education, economic development, and governance factors are stable across most of the models. Therefore, this research addresses a gap in the literature by diving into the core of confirmed factors. Thus, we look for the interconnections among education, economic performance, and governance effectiveness, and their collective influence on corruption perceptions, as well as for some time-lag evidence between determinants and the corruption perception.

Reflecting on the novelty that our study introduces to the literature on corruption assessment, at least a few aspects stand out. Firstly, our study appears to be a pioneering endeavor in conducting a comparative analysis of corruption perceptions across Schengen area nations, as far as our understanding extends. Our study departed from conventional methodologies by incorporating advanced statistical techniques alongside an interdisciplinary approach. The nuanced understanding of public perceptions of corruption stemmed from a theoretical framework that mixed various theories, including the bad apple theory, correlation theory, and organizational culture theory. Our innovative approach also centers on the analytical method, employing composite indicators that encapsulate the intricate aspects of education quality and governance effectiveness, mirroring public perceptions across various Schengen countries. These indicators transcend mere aggregated data, undergoing rigorous methodological treatment to furnish insights with heightened accuracy and relevance. Moreover, we offer a contemporary viewpoint by situating our analysis within the diverse economic landscape of the Schengen Area. This facilitates a more comprehensive grasp of the underlying underpinnings of corruption, thereby enhancing the value of our findings. Recognizing that comprehending corruption holds significant implications for early education in terms of averting future corrupt practices, integrating education closely with economic and governance issues actively contributes to enhancing interdisciplinary educational approaches. This can prove beneficial even in academic or professional spheres, fostering the development of practical skills. The paper strived to merge theoretical research with empirical analysis to find specific answers to two main research hypotheses:

**Hypothesis 1 (H1)**: Education, economic performance, and governance efficiency are interlinked in the broader context of sustainable development, with each potentially influencing corruption perceptions.
**Hypothesis 2 (H2)**: Education incentives, economic measured performance or perceived governance effectiveness enhance corruption perception in different Schengen countries.

The intricate interplay among education, economic success, and the efficiency of governance constitutes a cluster of variables capable of impacting how corruption is perceived. Hypothesis 1 posits that these variables are intertwined and collectively influence the prospects for sustainable development. It begins with the notion that education imparts both knowledge and principles that foster transparency, while strong economic performance may mitigate corruption. Additionally, effective governance has the potential to curb or, conversely, foster corrupt behaviors if it falls short. This hypothesis probes the ways in which these components shape public perceptions of corruption within the Schengen area.

Hypothesis 2 operates under the assumption that it’s not just the existence of educational incentives, economic performance, and the perceived effectiveness of governance that matter, but also the quality and interplay of these factors. It proposes that the perception and integration of these elements in various Schengen countries can greatly influence public perceptions of corruption. Enhanced educational programs emphasizing anti-corruption measures, evidence of economic growth, and noticeable governance efficacy can collectively serve as deterrents to corruption, molding public expectations and standards.

## 2. Theoretical background

### 2.1. Reflections on fraud and corruption

Fraud and corruption perceptions are two closely related concepts that influence and perpetuate each other in different ways. To understand this interconnectedness [14], it is essential to grasp how people perceive these phenomena then to explore how they interact. Understanding how people perceive fraud or corruption or pointing to their determinants is not an easy task, as previous research suggests. Such endeavor also needs calibration through perceptual theories and setting the anchors in the utility of empirical findings.

Fraud refers to any intentional act of deception for personal or professional gain. Fraud can take many forms, including, but not limited to, document forgery, tax evasion, shadow economy [15] and embezzlement. The hot topics of research on fraud have gained momentum in the areas of accounting and audit, highlighting the importance of further expanding interdisciplinary studies on fraud in accounting, organizations, and society [16,17].

Corruption has received various definitions over time. It may generally be perceived as an abuse of power entrusted to individuals for personal gain [18,19] Corruption includes practices such as bribery, nepotism, and favoritism. Unlike fraud, corruption often involves exchanges of favors between parties and can be more systemic.

In the complex dynamics of the professional environment, fraud and corruption are two interconnected phenomena that can seriously affect integrity [20,21] and effectiveness of control systems [22]. In corrupt environments, ethical and legal standards are often weakened, creating conditions ripe for fraud, as individuals may be tempted to exploit the vulnerability of the system for personal gain. Acts of fraud, such as document forgery, not only reflect this weakness, but can also exacerbate corruption by promoting bribery and nepotism [23].

This interaction is evident in the control mechanisms; both fraud and corruption can erode them, leading to a vicious cycle in which weakening control increases the sensitivity to further fraud and corruption incidents. Therefore, in order to ensure integrity and accountability, it is essential to implement robust control systems, including regular audits, transparent reporting procedures and strengthened legal frameworks [24].

In addition, organizational culture plays a significant role; organizations that do not value compliance and do not sanction fraud or corruption are at risk of becoming environments in which these negative behaviors not only flourish but can even be rewarded. In order to combat these phenomena, institutional reforms aimed at improving transparency and strengthening legislation are therefore crucial. Finally, education and awareness of the risks associated with fraud and corruption are essential to prevent them, discourage individuals from participating in such practices and strengthen organizational integrity [25].

In a study conducted within the context of Indonesian law, Suh [26] characterizes corruption as a crime that significantly impacts human rights, with the potential to disrupt the nation’s economy and impede its progress, leading to far-reaching consequences for future economic advancements. The combat against corruption is underscored as both a national and international priority, necessitating extraordinary efforts to combat this pervasive and systemic issue. This may entail the implementation of stricter regulations and penalties for corrupt activities, the promotion of transparency and accountability in governmental operations, and substantial investments in human capital development programs. Analyzing the interconnections among corruption, human capital, and unemployment within the Indonesian context, it has been observed that the enforcement of stringent anti-corruption laws and sanctions can foster a more conducive environment for economic growth and development, thereby enhancing the well-being of citizens [27].

As can be seen, the relationship between fraud and corruption control is complex and multidimensional. Although fraud is a symptom of a corrupt environment, it also facilitates and exacerbates corruption. To address these issues effectively, a holistic approach must be taken, including strengthening control mechanisms, promoting ethical organizational culture and supporting institutional reforms. Education and awareness-raising also play decisive roles in the prevention and combat of fraud and corruption.

### 2.2. Does education count for corruption assessment?

Education plays a pivotal role in the assessment and fight against corruption. In fact, it is one of the most effective means of building a more transparent and ethical society [28]. Education in integrity and ethics should begin at an early age and should continue throughout one’s life [29]. In schools, children can learn about the value of honesty and the adverse consequences of corruption. In higher education, young adults can participate in case studies and learn how to recognize and combat corruption in various fields [30,31]. Legal education can shape the importance of the rule of law and good governance for future lawyers and government officials [32,33]. Financial education greatly reduces the knowledge gaps of young entrepreneurs who strive to turn ideas into performant economic combinations, capable of transforming value added into profits and legal financial gains.

Through educational programs, individuals can learn about anti-corruption laws and how to apply them effectively [34,35]. In business, professional ethics education can prevent corporate corruption by training employees and managers in ethical practices and implementing compliance policies. Professionals such as accountants and lawyers can learn about the latest regulations and avoid involvement in corrupt acts by continuing their education.

Education on anti-corruption becomes imperative for human resource training and character building, serving as a fundamental requirement for effective human resource development. Corruption, stemming from diverse internal and external factors, demands a comprehensive and exceptional system for its eradication, which entails fostering an anti-corruption culture within society as a preventative measure. In the economic domain of European nations, the significance of proactive legal frameworks and the implementation of measures promoting comprehension and enforcement actively bolster economic development [36].

At the government level, training programs can help public officials better understand the risks and consequences of corruption and promote transparency and accountability [37]. Furthermore, civic education empowers citizens to recognize and report corruption, strengthening civil society and supporting anti-corruption efforts [38]. Technology and innovation in education can expand access to information and provide platforms for education and civic engagement. For instance, massive open online courses can provide large-scale ethics and anti-corruption training.

The influence of corruption on the quality of education and the development of human capital is significant, underscoring the necessity for a comprehensive understanding of the factors that govern and the role of human capital in fostering economic growth and development [39]. Investment in human resources and education is essential for fostering innovation and economic advancement, with research indicating a positive correlation between human capital and economic growth [40]. Likewise, in related studies, innovation has been linked to mitigating the adverse impacts of corruption on economic growth, particularly in developed nations [41].

In summary, education is a vital tool for assessing and combating corruption. Education can contribute to building stronger society’s institutions and culture of integrity by encouraging critical thinking, promoting ethical values, and providing specific knowledge.

### 2.3. Economic performance and governance effectiveness perceptions relate to corruption

Economic performance and the perception of governance effectiveness are often linked to the level of corruption within a state. In high-performing economies, where governance is perceived effective, corruption tends to be less. This is due to several factors, including a solid judicial system, a transparent public sector, and access to information.

Anti-corruption entities hold a crucial position in governance, directly impacting foreign investment and the economic competitiveness of developing nations. Conducting a study on corruption’s detrimental effects on economic growth within the Economic Community of West African States (ECOWAS), Mohammed et al. [42] noted that one of corruption’s notable consequences is its tendency to diminish economic growth.

Numerous scholars have investigated the adverse effects of corruption on society, emphasizing its destructive potential to undermine governmental structures, posing a significant barrier to governance and development. Combatting corruption necessitates active engagement from the government and its institutions, fostering an anti-corruption ethos and raising public awareness about the perils and detrimental effects of corruption. Corruption affects behavior, eliciting unexpected responses, preferences, or aversions toward various political ideologies, irrespective of individuals’ educational backgrounds [43].

Corruption undermines economic growth and development by distorting competition and the inefficient allocation of resources [44]. Foreign investments can be discouraged by a corrupt business climate, and investors’ confidence diminishes when governance is perceived as ineffective. Furthermore, corruption can lead to tax evasion, a reduction in state income, and a limitation of government capacity to provide essential public services.

On the other hand, effective governance promotes a robust economy by creating a stable environment for businesses and ensuring fair and consistent application of law [45]. This encourages entrepreneurialism and can lead to a more equitable distribution of wealth. Transparency in governance reduces corruption opportunities, and independent justice ensures that convicted offenders are held accountable.

Public education and awareness of corruption are essential to building a culture of integrity [46]. Informed people are less likely to tolerate corruption and are more likely to demand accountability. Furthermore, in an effective state system, public feedback mechanisms and civic participation can contribute to the creation of policies that better reflect the needs of citizens.

In a virtuous cycle, economic growth can finance improvements in public services, increasing governance efficiency [47] and reducing corruption. For example, improving infrastructure can reduce transaction costs and improve the economy’s competitiveness, reducing corruption and stimulating economic performance.

In conclusion, there is a close relationship between economic performance, perception of the effectiveness of governance, and corruption. Effective and transparent governance can improve economic performance, and strong economies can strengthen governance and reduce corruption. A multifaceted approach, including legislative reforms, institutional strengthening, education, and civic engagement is necessary to combat corruption and promote effective governance and robust economic performance.

## 3. Data sources and research methods

### 3.1. Description of data

Data matrix is formed from N = 14 countries and the follow-up period are 2003–2022, thus T = 20 years. The selection of countries accounted for European countries included in the Schengen area, for which reliable and comparable data could be gathered: Austria, Czech Republic, Denmark, Finland, France, Germany, Hungary, Italy, Netherlands, Poland, Portugal, Slovenia, Spain, Sweden. The Schengen area is a zone where member countries set common judicial policies, abolished their internal borders for free movement of people and implemented common rules for mitigating fraud risks and fighting criminality. Acknowledging that such member countries rely on common policy safeguarding values, we observed that the public’s perception on fraud and corruption differed, thus decided to deepen the understanding on probable factors or causes.

The overarching concern regarding the implementation of effective policies aimed at achieving sustainable development objectives has spurred exploration into new avenues of research. Among these, the perception of corruption assumes a pivotal role, impacting trust in governance, the efficacy of economic structures as well as social awareness of the corruption perspective. Yuan and Ishak [48] examined the correlation among corruption, governance, and economic growth across several ASEAN countries (Singapore, Malaysia, Thailand, Indonesia, and the Philippines), and revealed intriguing associations between corruption and gross domestic product per capita. In a similar vein, Awan et al. [49] exposed noteworthy connections among corruption, governance, and economic growth in a selection of SAARC countries (Bangladesh, India, Nepal, Pakistan and Sri-Lanka), pointing to corruption as a hindrance to economic development. The correlation between economic growth and corruption has been extensively explored. Past research consistently supports the notion that corruption inherently impedes economic growth [45]. Conversely, alternative studies support the "greasing the wheels" hypothesis, suggesting that corruption may, in specific contexts, facilitate economic growth [5]. Agerberg [43] explored the interplay of corruption and education, emphasizing political attitudes and democratic behavior, and observed that corruption perception may not always be determined by education levels. Building upon prior research, our study delves deeper into exploring the connections between corruption, governance, economic performance, and education, aiming to provide a more comprehensive understanding of the interrelations among these crucial factors.

Our proposed approach investigated how education, economics, and governance influence corruption. Current analysis dealt with an initial set of 13 variables categorized into two broad categories: (a) the dependent variable, CPI, reflected the corruption perception index, and (b) independent variables: EduIndex accounted for education assessment, GNI per capita accounted for economic performance, GovIndex indicated the general perception of local governance. The sample data was collected from various reliable sources, such as Eurostat, the World Bank and Trading Economics.

CPI is a composite indicator that includes data on the general perception of corruption by the local population, in the sense of the misuse of public power for personal gain, thus harming society. The CPI is measured between 0 and 100, where the 100 mark indicates a country with a very low level of corruption, while the 0 mark indicates a highly corrupt country. The CPI component includes the perception of irregularities in public funding management, irregular tax collection, biased public procurement, irregularities in judicial acts, irregularities in exports or imports, perceived levels of corruption in the public administration or in its relationship to the local business environment [50].

Regarding the independent variables, the study further investigated a number of 11 variables classified into two composite variables [51,52] as shown in Table 1.

**Table 1:** Description of variables.

Independent variables Abbreviation	Description	Measurement
**EduIndex**	See Eq (1)	
NEET 15_29 (%)	Young people aged 15–29 who are not employed, enrolled in a school or other training program	% of total youth 15–29 of age
EduTerf	Current expenditures are consumed in the current year and, if necessary, must be renewed in the following year. It includes staff compensation and current expenditures other than for staff compensation (e.g., teaching materials, ancillary services, and administration).	% of the direct expenditure of public educational institutions (instructional and non-instructional). Financial assistance for students and other transfers are excluded from direct expenditure.
ATG25	The percentage of the population aged 25 and over who have achieved or completed short-term higher education.	% of total population aged 25 or over
PubEdu	Public expenditure on education takes into account the level of education and the orientation of the program.	% of GDP (Gross Domestic Product) attributed to education
**GNI**	Gross National Income is defined as gross domestic product plus foreign net revenues from employee compensation, property income and net taxes minus production subsidies. Compensation for employee’s receivable from abroad is that earned by residents who live mainly in the economic territory but work abroad, or for people who live and work abroad for short periods (seasonal workers) and whose center of economic interest remains in their home country.	$/person
**GovIndex**	See Eq (2)	
CtrCor	Control of corruption captures the perceived extent of acts of public power directed towards private gain, including petty and grand forms of corruption.	Values between -2,5 and 2,5
GovEff	Government Effectiveness: Estimate—captures perception of public services quality and its independence from political pressures, the quality of policy formulation and implementation, and the credibility of the government’s commitment to such policies.	Values between -2,5 and 2,5
RegQual	Regulatory Quality: Estimate—measures the perception of government capability to formulate and strengthen sound policies and regulations that enable and promote the development of the private sector.	Values between -2,5 and 2,5
PolStab	Political Stability and Absence of Violence/Terrorism: Estimate—measures perceptions of the likelihood of political instability and/or political violence, including terrorism.	Values between -2,5 and 2,5
Rule_Law	The rule of law encompasses perceptions of the degree to which agents trust and comply with the rules of society, especially the quality of contract execution, property rights, police and courts, and the likelihood of crime and violence.	Values between -2,5 and 2,5
UnRate	Unemployment rate–the rate of unemployment out of the total working population	% of total employable population
VoiceAc	Voice and Accountability: Estimate—reflects perceptions of the extent to which citizens of a country can participate in the election of their governments, as well as freedom of expression, freedom of association and freedom of media.	Values between -2,5 and 2,5

The construction of EduIndex and GovIndex involves aggregating multiple variables associated with education and governance, respectively, through weighted sums as outlined in Eqs (3) and (4). Each variable accounted in the equations is detailed in Table 1 and represents distinct facets of education and governance. The coefficients in Eqs (3) and (4), denoted as *a*_*1*_ to *a*_*4*_ for EduIndex and *b*_*1*_ to *b*_*6*_ for GovIndex, signify the weights assigned to each variable in the corresponding index. The composite indexes are determined based on the perceived significance of individual variables in capturing diverse dimensions of education and governance. Subsequently, the weighted values of each variable are combined to yield the overall EduIndex and GovIndex, offering a comprehensive assessment of various aspects within education and governance, respectively.

### 3.2. Research framework

**Fig 1:**
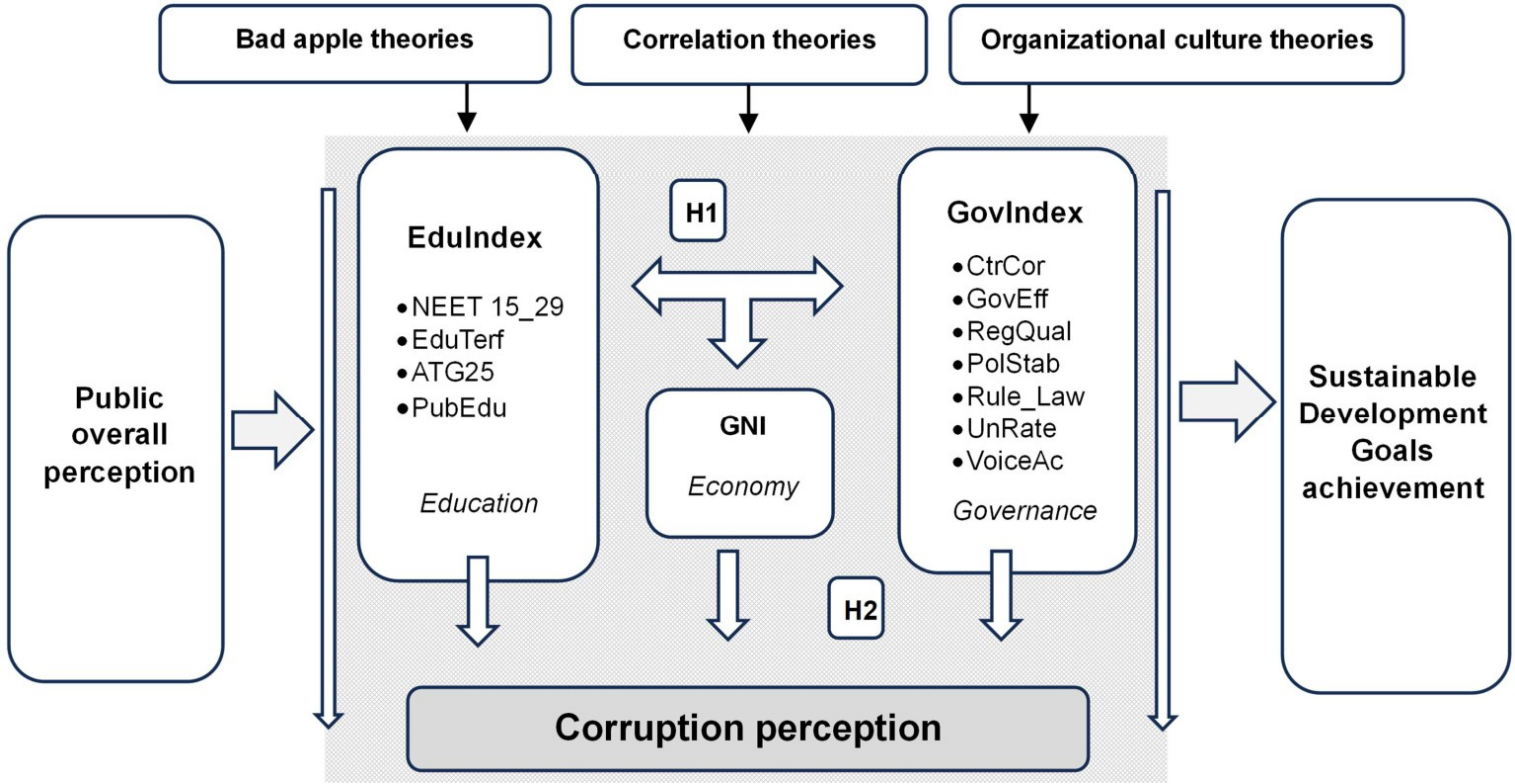
Investigation of corruption perceptions within the framework of education, economic performance, and governance.

As Fig 1 shows, data corpus consists of a set of composite indicators that support our research, drawing relationships between them and substantiating significant findings of academic and empirical value for the determinants that trigger corruption perceptions.

The first hypothesis tested by this paper (H1) was whether education, economic performance, and governance efficiency are interlinked in the broader context of sustainable development, with each potentially influencing corruption perceptions.

Taking into account previous research, the paper acknowledged that overall public perception may be influenced by several contexts. Building on the approach of De Graaf [53], multi-theoretical perspective has been embedded in our analysis to address the intricacies of the research flow presented in Fig 1. Consequently, the analysis of perceived causes of corruption was calibrated by bad apple theories, correlation theories and organizational culture theories.

Bad apple theory refers to a theoretical approach to corruption outcomes triggered by causal faulty individual integrity chain reactions. If morally flawed individuals are regarded as” bad apples” for determining corrupt behaviors, then the bad apple theory would suggest that corruption is most likely caused by the dishonest values of officials, public authorities, decision makers or governance actors. As Graaf noted, people tend to act based on their moral principles or values, so consequently corruption may be perceived as an effect of wrongful or” bad” human values [54].

Correlation theories did not emerge from specific causal models but tended to study contextual correlations and interconnections between different conditions or situations [55]. Correlation theories tend to ignore contingencies and focus on variable investigation, bringing the empirical dimension closer to the theory.

A different theoretical approach to perception of corruption focuses on organizational culture and structure rather than individual corrupt agents. The theory of organizational culture relies on organizational values and group culture that are deemed to influence work behavior and lead to a particular mental state or behavioral predilection within the group. As Punch [56] noted, individuals seeking personal gains observed in authority institutions are likely to be markers of extended deviations in group dynamics, as group behavior is rooted in certain cultural or structural organizational arrangements [57,58].

Such theories have incited research into corruption control and combat and have stimulated debate about the contextual need for research into the factors of corruption that ultimately lead to organizational culture alteration [59–61] or negative changes in society [62].

The theoretical analyses of this study are anchored in both empirical economic constructs and qualitative measurements of public perception in order to thoroughly deepen the understanding of the observed incidence of corruption. The second hypothesis (H2) examined to what extent education incentives, economic measurement results or perceived effectiveness of governance would enhance the perception of corruption in different Schengen countries, based on panel data methods. The results pointed to very interesting findings, as discussed in the following sections of the paper.

### 3.3. Research methods

**Fig 2:**
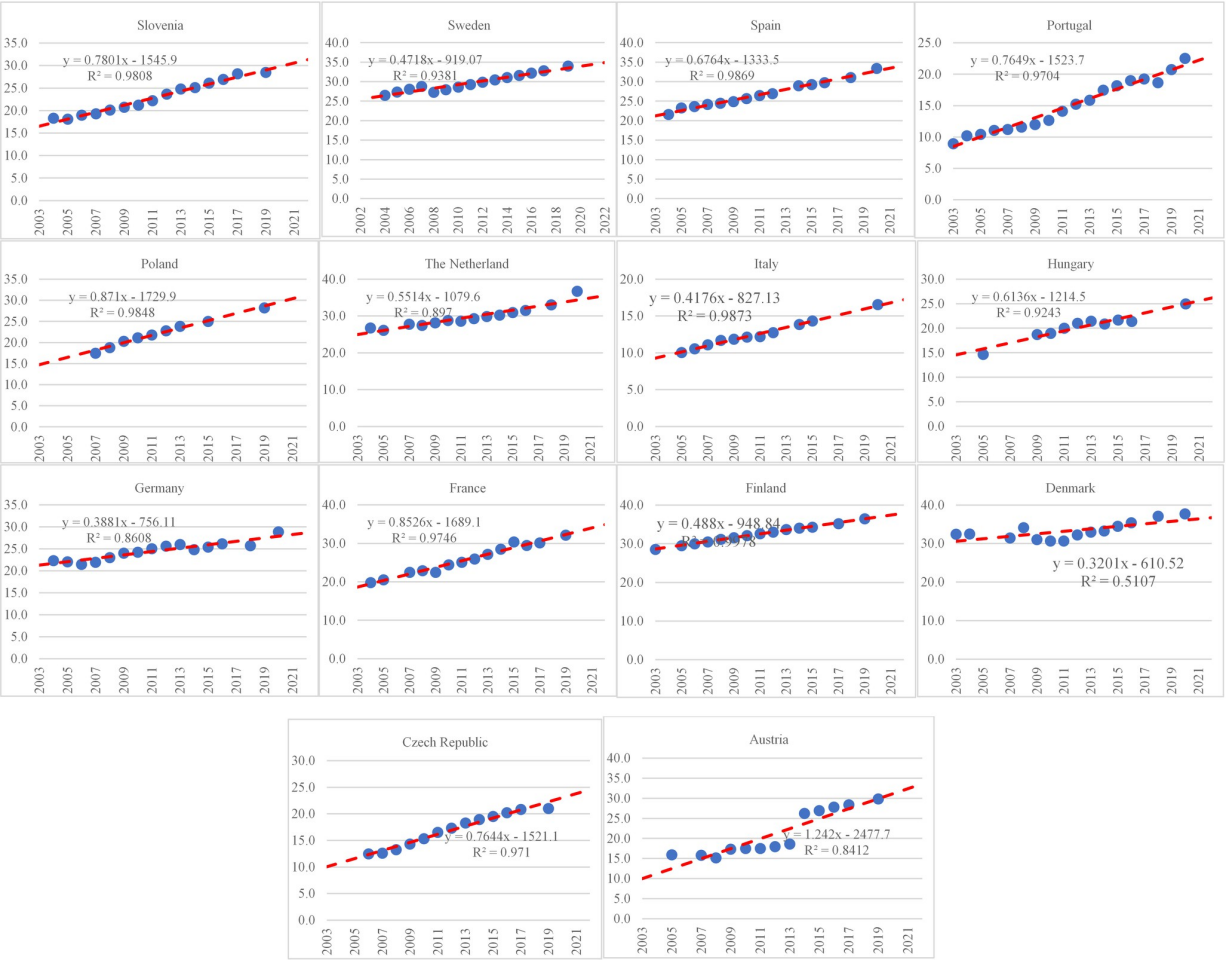
ATG24 adjustments.

**Fig 3:**
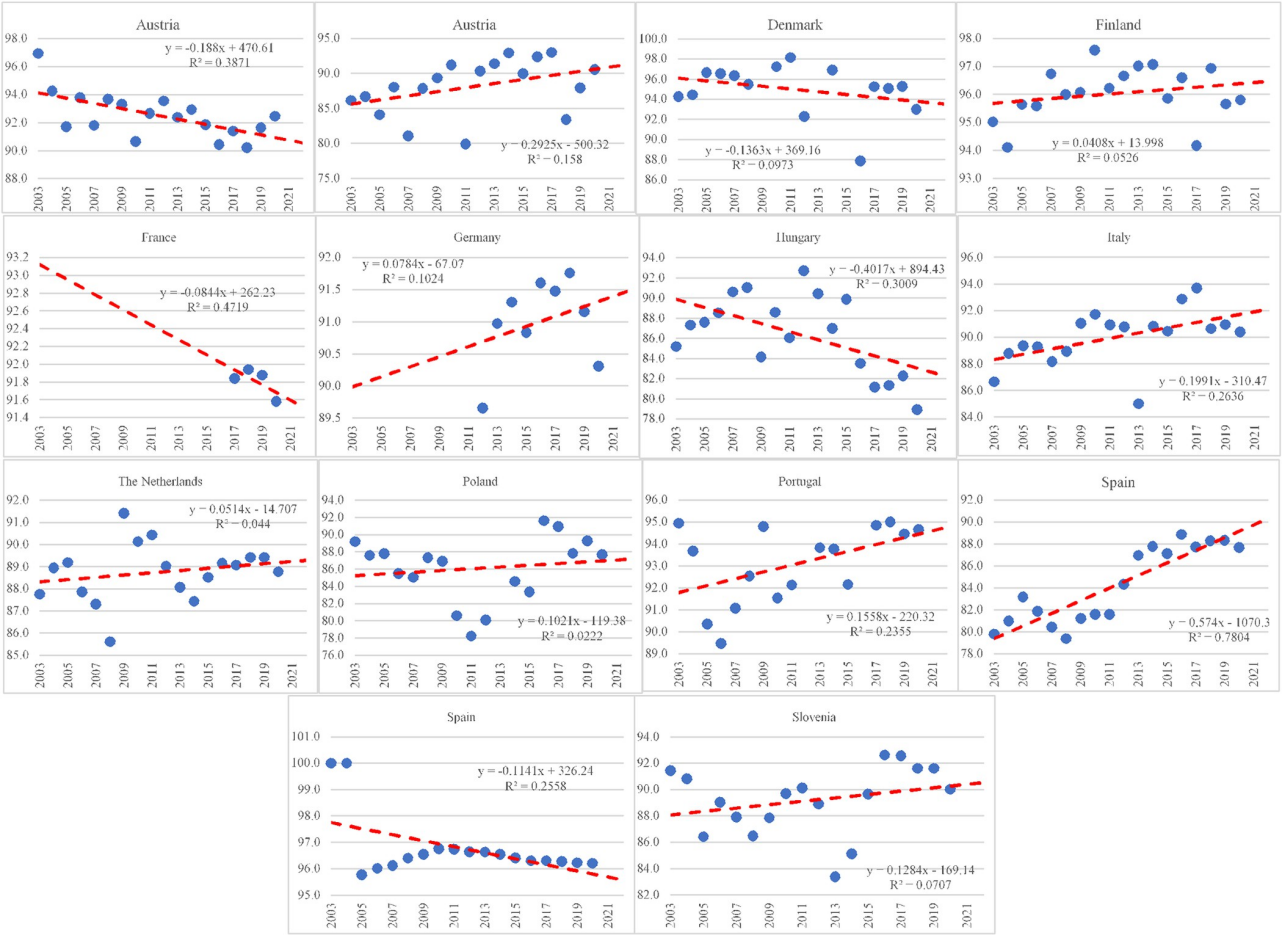
EduTerf adjustments.

We recall that the main objective of this study is to see the impact of potential drivers of corruption. One issue is that EduTerf and ATG25 and PubEdu variables contain several missing data. Using simple linear regression models, we input missing data. Main aspects about regression models are presented in Appendix A, Figs 2 and 3. Another issue is that the list of 13 variables which share similar contents present high variation and large correlation among them. The descriptive analysis presents the main results and give us valuable insights about the appropriate steps to follow. In consequence, we apply principal component analysis to extract the core information.

Following Jolliffe and Cadima [63], we recall that in the case of PCA method we start from a matrix of k factors, *X*_1_,…*X*_*k*_, each with a dimension of n*T, where n = number of countries and T = number of periods. We search to retain *p*≤*k* components, formed as linear combination of the existent *X*_*i*_, i = 1…k, with the goal to exctract the maximum variation from the initial data. The maximization goal, under the constraint of *c*^*t*^*c* = 1, drives us to a classic Lagrangean problem, which may be defined like in the relation (1). Due to different scales of initial variables, the computational steps are performed on the standardized (Z-scores) form of the initial variables.

$$\max\left(var(\sum_{i=1}^{k}c_{i}Z_{i})\right)=max(var(Zc)$$ (1)

or If we assume that var-covar matrix of the k variables is *S*_*z*_, then the problem becomes $max(c^{t}S_{z}c-\lambda (c^{t}c-1))$

Applying first derivative, taking the vector of coefficients, c, as unknown, we have the equation:

$$S_{z}c-\lambda c=0$$ (2)

The *λ* vector contains the Langrange multipliers and aswell they represent the eigenvalues of the symmetrical matrix *S*_*z*_. The coefficients c represents the eigenvector corresponding to each eigenvalue *λ*.

The next problem to solve is the optimal number of principal components and to assess the adequacy of PCA method. New goals defined by the selection of components and performance of the method, are achieved taking into account both statistical and practical aspects. Eigenvalues above a referenced value, usually unit, the scree plot, or the Kaiser-Meier-Olkin (KMO) are the most used methods. To support the statistical decision, we recall that on the principal diagonal of the matrix *S*_*z*_ we have the variances of each initial variables, Z, by conservation of the information we have that $Tr(S_{z})=\sum_{i=1}^{k}\lambda_{i}$. One may select p components which retain the maximum of the initial variance, by evaluating the performance of the $\mathrm{sum}\sum_{i=1}^{p}\frac{\lambda_{i}}{Tr(S_{z})}$, or looking for those *λ*_*i*_ > 0.7. Meanwhile, many authors like Jolliffe and Cadima [63] or XX consider that PCA is a suitable technique when this sum is above 0.7 for few components. Others like Kherif and Latypova [64] prefer more restrictive condition and rise the threshold to 0.8. We recall that we search to explain the corruption drivers by different aspects. Then, we look to accomplish the statistical aspects as much as possible, but to retain, if possible, one component from each domain. In such manner we aim to build a component named EduIndex as a linear combination of NEET, EduTerf, PubEdu and ATG25, following the relation (3):

$$EduIndex=a_{1}*NEET+a_{2}*EduTerf+a_{3}*ATG25+a_{4}*PubEdu$$ (3)

Using the same method, we construct the GovIndex as a linear combination of GovEff, RegQual, PolStab and VoiceAc, thus:

$$GovIndex=b_{1}*CtrCor+b_{2}*GovEff+b_{3}*RegQual+b_{4}*PolStab+b_{5}*RuleLaw+b_{6}*UnRate+b_{7}*VoiceAc$$ (4)

We present the achieved results in paragraph 4.1.

#### 3.3.1. The assessment of stationarity via panel unit root test

With the help of the initial and synthetized variables we specify a panel regression model. The dimension of the data set and the expected behavior of the variables allows us to test few panel options. Based on the assumption that in T = 20 years, the socio-economic indicators share some trends, first we employ Im-Pesaran-Shin (IPS) panel unit root tests for a quantitative assessment of the stationarity [65], for each variable. We recall that the IPS test for finite panel data structure starts from the equation:

$${\Delta y}_{it}=\alpha_{i}+\beta_{i}y_{i,t-1}+\sum_{j=1}^{pi\leq 4}\rho_{ij}{\Delta y}_{i,t-j}+\varepsilon_{it}$$ (5)

Where *α*_*i*_ = constant for each of 14 cross-sections, *β*_*i*_ = autoregressive coefficients, *p*_*i*_ = maximum lag selected based on the minimization Akaike Information Criteria (AIC), y = the variable studied ∈{CPI, GovIndex, EduIndex, GNI}, *ε*_*it*_ = residual component. The j index counts for lags, the i index varies by cross-section and t is the period-index. The test verifies the hypotheses:

H0: all *β*_*i*_ = 0, thus the variable y has unit root for all cross-sections.

H1: at least one *β*_*i*_<0, at least one cross-section is stationary in the sense of y variable.

According to Im et al. [65], the specific test, in normal cases is the averaged value of the country individual t-statistic (*t*_*iT*_) used to assess the hypothesis *β*_*i*_ = 0 like defined above. If the $\bar{t}$ values fall above the critical value given by the authors, then we reject the hypothesis of unit root. But, in the case of heterogenous panels following the case of Im et al. [65] we apply the following statistics:

$$W\bar{t}(p,\rho )=\frac{{\sqrt{14}\{(\bar{t}(14,T)-(\frac{1}{14})\sum_{i=1}^{N}E[t_{iT}(p_{i},0)|\beta_{i}=0]\}}_{T,14}}{{\left(\frac{1}{14}\sum_{i=1}^{14}var(t_{iT}(p_{i},0)|\beta_{i}=0]\right)}^{0.5}}∼N(0,1)$$ (6)

Based on the overall unit-root tests results, and assuming heterogenous results by cross-section, we define several models presented in the next sub-paragraphs.

#### 3.3.2. Specification, hypothesis, estimation, and validation of different econometric models

Assuming that corruption has some country-specific fixed factors, first, and the stationarity of variables, we employ a static model fixed-effects (FE) model:

$$M1:\;{\Delta lnCPI}_{it}=\beta_{0i}+\beta_{1}*{\Delta EduIndex}_{it}+\beta_{2}*{\Delta GovIndex}_{it}+\beta_{3}*{\Delta LnGNI}_{it}+u_{it}$$ (7)

Where *u*_*it*_ is the idiosyncratic error term, *Δ* is the first-difference operator, i and t are two indexes for N cross-sectional and T time units. It is well-known that for Panel-FE model, *u*_*it*_ must be independent of covariates *X*_*it*_, and specific factors *β*_0*i*_. Also, the multicollinearity effects among predictors are negligible, the residuals are from a known distribution with zero mean and the outliers are missing [66]. Assuming the existence of trend both in the dependent variable and its regressor, and that we have no additional information about *β*_0*i*_, if they are truly fixed in time, first we use only the first differenced model, M1, skipping thus the initial form [67]. Then, we estimate M1 model with and without drift. In this case we assume that we capture some specific factors of the countries which remain constant in time. In the case of FE model, we also ignore a potential dynamic association of corruption measure. Validation of the model is made with the help of Fisher Validity test and Panel Durbin-Watson statistics for serial autocorrelation, as defined for example in Baltagi [68].

To correct this limitation following Baltagi [68] who notes that Panel with Generalized Method of Moments (GMM) and Panel FE are consistent when T/N is constant, we propose also Multifactorial Dynamic Panel model with GMM estimation. In this case we test two types of models:

M2a:

$${lnCPI}_{it}={\sum_{s=1}^{\leq 3}\alpha_{s}lnCPI}_{it-s}{+\sum_{s=0}^{\leq 3}\beta_{s}lnGNI}_{it-s}+{\sum_{s=0}^{\leq 3}\lambda_{s}EduIndex}_{it-s}{+\sum_{s=0}^{\leq 3}\theta_{s}GovIndex}_{it-s}+\sum_{i=1}^{T-1}d_{i}t+u_{it}$$ (8)

This class of model includes an expected dynamic relation of corruption perception, thus:

M2b:

$${\Delta lnCPI}_{it}={\sum_{s=1}^{\leq 3}\alpha_{s}\Delta lnCPI}_{it-s}{+\sum_{s=0}^{\leq 3}\beta_{s}\Delta lnGNI}_{it-s}+{\sum_{s=0}^{\leq 3}\lambda_{s}\Delta EduIndex}_{it-s}{+\sum_{s=0}^{\leq 3}\theta_{s}\Delta GovIndex}_{it-s}+\sum_{i=1}^{T-1}d_{i}t+w_{it}$$ (9)

Where *u*_*it*_ = *v*_*i*_+*ε*_*it*_ and *w*_*it*_ = *Δu*_*it*_, with *v*_*i*_ = country specific error term. One can notice that by adding *Δ* operator in the M2 class models, the cross-sectional error vanishes its effects. The letters s accounts for 1,2, to maximum 3 time-lags, and the *d*_*i*_ are dummy variables which accounts for time effects, assuming that in the non-differenced model, the time effects are rather linear than constant. As described also in Bond et al. [69], the hypotheses of both models impose that all residuals involved in the models have zero mean, and *ε*_*it*_ and respectively *w*_*it*_ are serially uncorrelated. Also, the specific factors *v*_*i*_ are independent of *ε*_*it*_. Furthermore, in both models the covariates GNI, EduIndex and GovIndex and their first differenced values should be also no correlated with disturbance component *u*_*it*_ and *Δu*_*it*_. These conditions are additionally to the initial well-known [70] conditions with further developments like synthetized in [71], which impose that for all countries, *ε*_*it*_ are uncorrelated with the initial *lnCPI*_*i*0_ and respectively *ΔlnCPI*_*i*0_, for all periods. Also, for both class of M2 models, all the lagged values of the CPI included as regressors must be uncorrelated with the residuals. An extended form of the estimator applied for M2a and M2b models, with the help of W instrument matrix, may be found in various sources, like in Baltagi [68].

For each case, specific tests like t-tests and Fisher tests are applied to verify the coefficient significance and model overall significance. Also, the Sargan-Hansen Test is applied to check the validity of instruments in the GMM technique.

Data formatting and data input was performed with the help of Microsoft EXCEL. Descriptive analysis, PCA and panel model estimations were performed in R-Studio v4.3.1 following the method described in Fritsch et. al [71] and with the help of multiple R-libraries, mentioned in Appendix B. The achieved results are presented in the next section.

## 4. Results and discussion

### 4.1. Hypothesis 1 results obtained with descriptive analysis and principal component analysis

**Fig 4:**
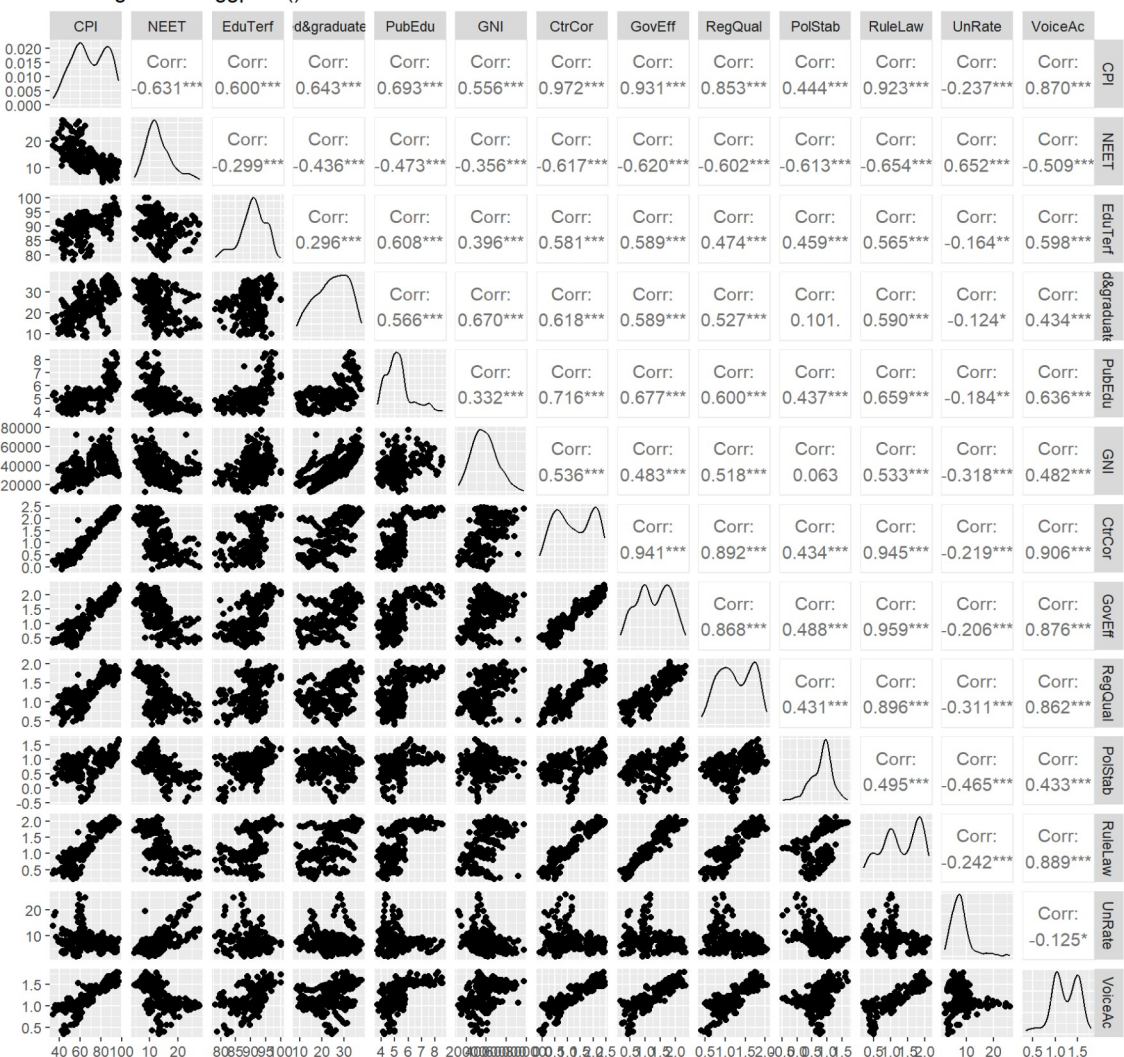
Scatterplot, distribution and Pearson correlation matrix of the initial variables.

Following the values and the shapes presented in Fig 4, we observe high values of the Pearson correlation coefficient. Furthermore, all correlations are marked as statistically significant, accepting type I error lower than the 0.05 threshold. The empirical distributions of each variable suggest various behaviors, with a mix of uni and bi-modal shapes and also with some heavy-tails distributions. As expected, due to these characteristics, a dimensional reduction technique, such as Principal Component Analysis based on non-parametric measures may be a good solution.

**Fig 5:**
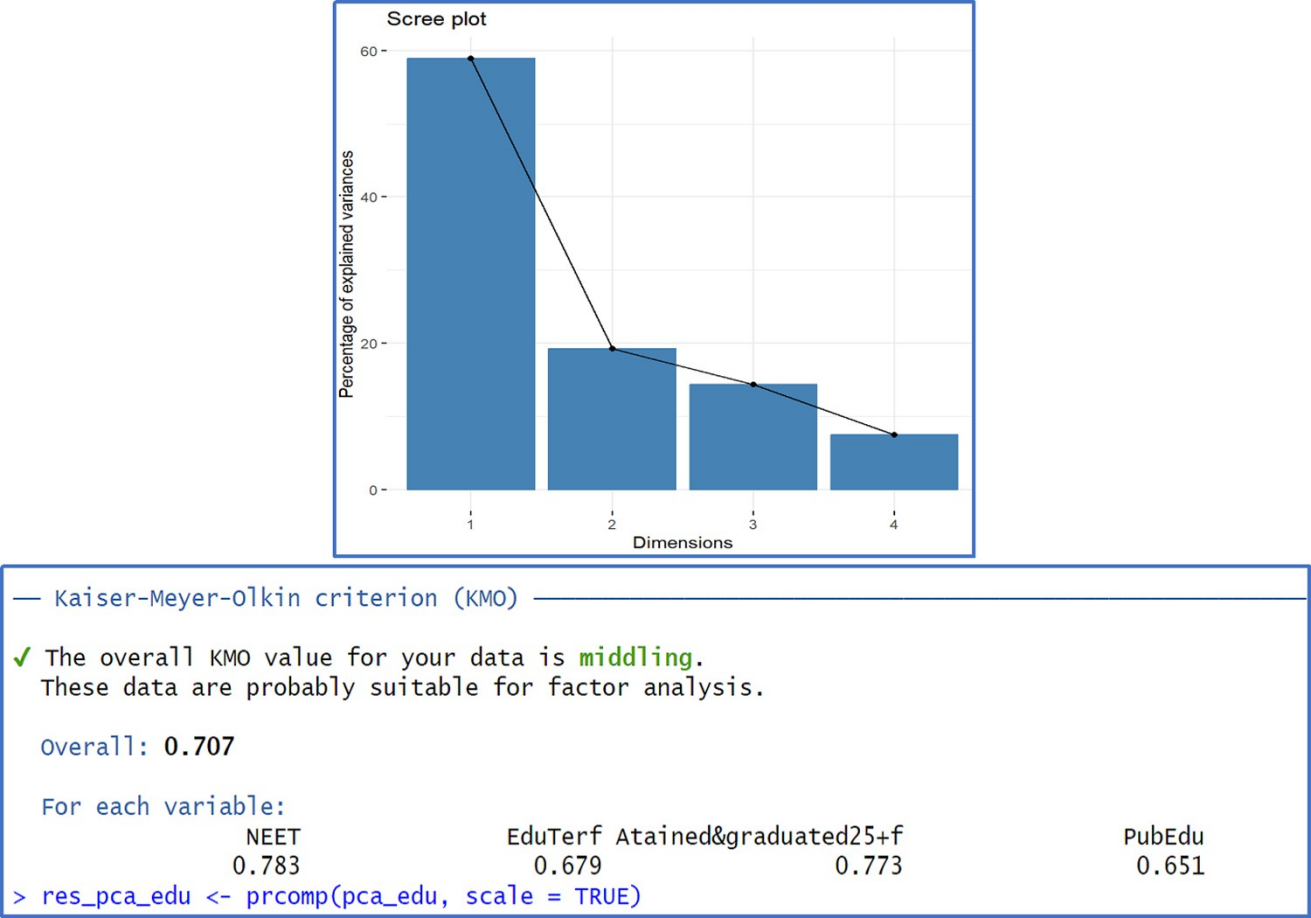
PCA results for Edu Index.

**Fig 6:**
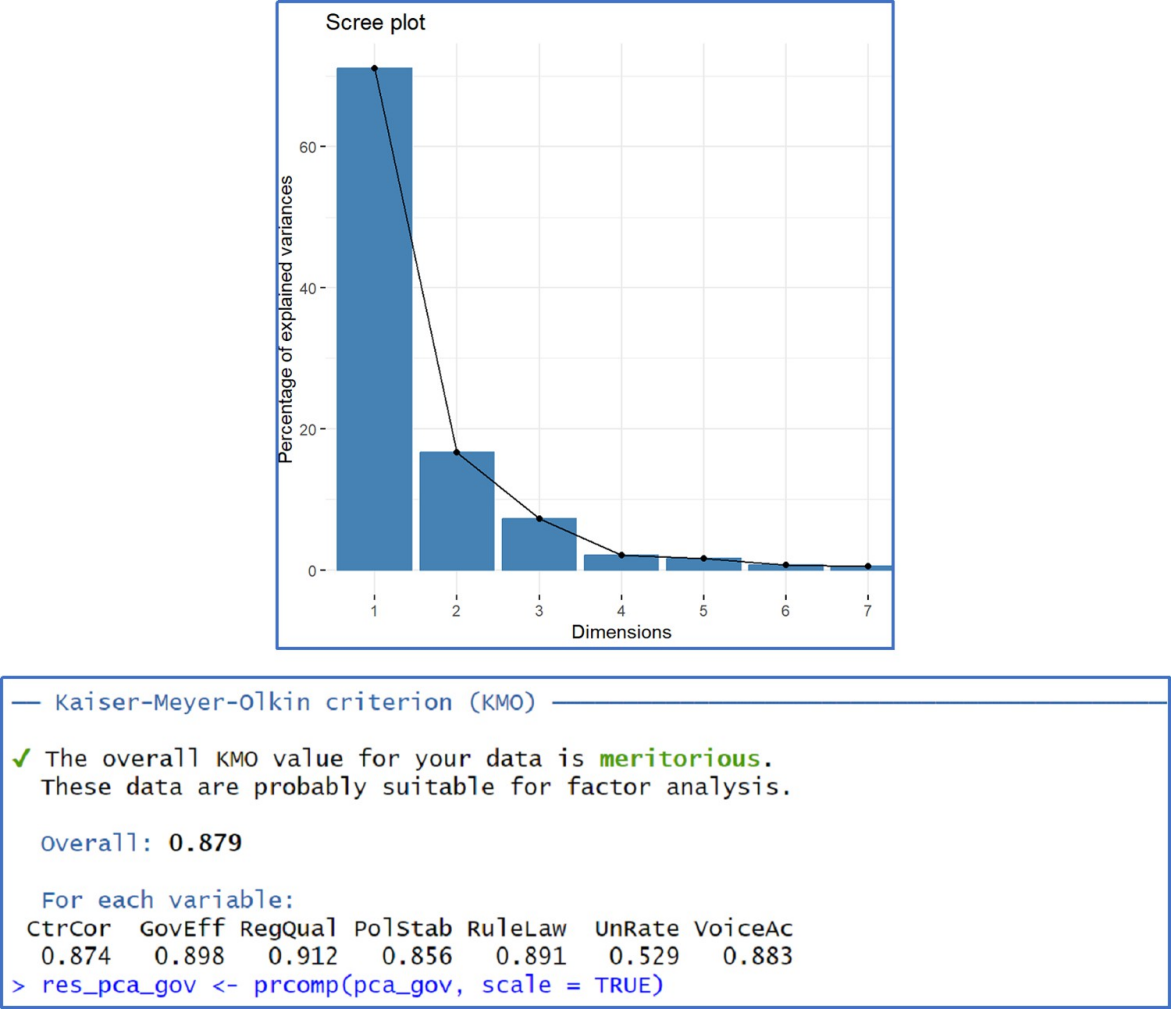
PCA results for GovIndex.

Applying PCA for Edu variables based on Spearman rank correlation, the KMO index is 0.707 suggesting a moderate match of the method. The first PC extracted retains near 60% of total variance. On the government capabilities we observe that First PC extracts 71% and the overall KMO index is 0.879. Therefore, in this case data source has a very good adequacy to PCA method. One may find additional information regarding PCA results in Appendix B, Figs 5 and 6.

The high percentage of variance explained by the first PCs may also suggest that while the economic indicators used are diverse, they may be driven by common underlying economic trends or policies that are influential across the Schengen Zone. This could reflect the interconnectedness of educational outcomes and government efficiency with broader economic conditions such as investment in human capital, regulatory quality, and institutional performance.

### 4.2. Hypothesis 2 results obtained with panel analysis

**Fig 7:**
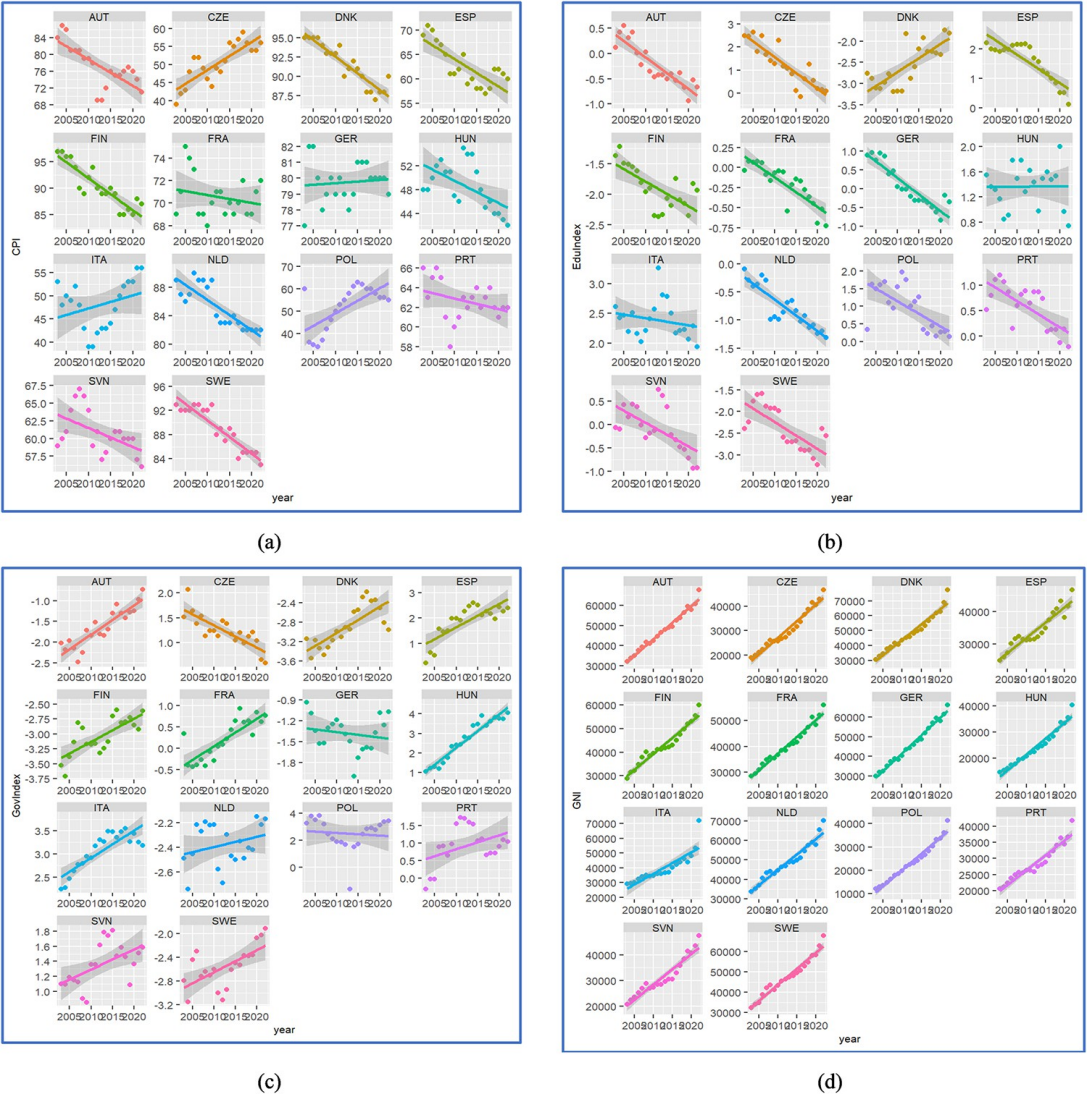
The dynamic of main variables: (a) CPI; (b) EduIndex; (c) GovIndex; (d) GNI.

Performing the analysis of the initial and derived variables several interesting findings were obtained. As Fig 7 underlines, various trends characterize each variable by country. An increase of CPI index can be noticed for Czech Republic, Poland and Italy, a rather stationary behavior is in Germany, while in the rest of the countries the slope is negative. The composite Index of Education factor has mostly negative slopes. One positive trend capture attention in Denmark. The Government Index also has mixed positive-negative values. The GNI per capita has only positive slopes in each country.

Overall, the results show heterogeneity between countries and probably mixed stationarity. Therefore, first we employ IPS test, and we analyze the stationarity. First difference transformation may solve the problems related to autocorrelation. Tables 2A-2D describe the results for IPS unit root for each variable and country, as-well as the aggregate decision.

**Table 2:** a. Unit root analysis for CPI and its derivates. b. Unit root analysis for GNI and its derivates. c. Unit root results for EduIndex and GovIndex and their derivates.

Country	CPI			LnCPI			ΔLnCPI		
	pi	*t*(*p*_*i*_, *ρ*_*i*_)	p-value	pi	*t*(*p*_*i*_, *ρ*_*i*_)	p-value	Pi	*t*(*p*_*i*_, *ρ*_*i*_)	p-value
1	1	-2.84	0.181	1	-2.85	0.180	0	-3.84	0.014
2	3	-5.27	***	3	-5.47	**	0	-3.64	0.027
3	0	-2.82	0.186	0	-2.79	0.20	0	-5.07	**
4	1	-4.25	***	1	-4.29	**	1	-5.17	**
5	1	-3.47	0.043	1	-3.45	0.044	4	-5.69	**
6	0	-4.21	**	0	-4.24	**	1	-5.24	**
7	0	-2.21	0.48	0	-2.16	0.50	0	-4.51	**
8	1	-1.23	0.90	1	-1.39	0.86	1	-2.92	0.15
9	0	-2.31	0.43	0	-2.30	0.43	0	-4.53	**
10	0	-3.72	0.02	0	-3.76	0.018	0	-13.69	***
11	3	-3.38	0.05	3	-3.36	0.056	0	-4.27	**
12	0	-1.83	0.68	0	-1.87	0.066	2	-4.09	**
13	0	-3.44	0.046	0	-3.43	0.046	0	-6.60	***
14	4	-5.61	***	4	-5.57	***	0	-4.31	**
Individual intercepts and trend included in the equation									
$W\bar{t}$ = -4.75, p<0.01				$W\bar{t}$ = -4.85, p<0.01			$W\bar{t}$ = -12.20, p<0.01		

Country	GNI			LnGNI			ΔLnGNI		
	pi	*t*(*p*_*i*_, *ρ*_*i*_)	p-value	pi	*t*(*p*_*i*_, *ρ*_*i*_)	p-value	Pi	*t*(*p*_*i*_, *ρ*_*i*_)	p-value
1	2	-0.97	0.95	3	-4.61	***	4	-5.77	***
2	0	-0.65	0.98	1	-2.64	0.26	2	-2.91	0.15
3	0	1.71	0.99	0	-1.77	0.71	1	-1.72	0.74
4	0	-0.53	0.98	0	-2.20	0.49	1	-2.79	0.21
5	3	-1.69	0.76	0	-3.43	0.048	1	-4.32	**
6	0	-2.52	0.32	3	-4.96	**	4	-5.82	***
7	2	2.12	0.99	4	-2.47	0.34	1	-5.59	***
8	4	3.20	1	3	1.34	0.99	2	-1.97	0.62
9	0	-0.75	0.97	0	-2.04	0.58	1	-3.30	0.066
10	2	2.90	1	4	-4.27	**	3	-0.80	0.96
11	3	-1.54	0.81	3	-2.51	0.32	1	-3.56	0.033
12	0	-2.23	0.47	0	-2.44	0.36	0	-3.77	0.018
13	0	-0.82	0.96	0	-2.36	0.40	2	-3.28	0.068
14	0	-0.28	0.99	0	-1.41	0.86	3	-3.16	0.092
Individual intercepts and trend included in the equation									
$W\bar{t}$ = 7.56, p<0.01				$W\bar{t}$ = -1.84, p = 0.033			$W\bar{t}$ = -5.08, p<0.01		

Country	EduIndex			ΔEduIndex			GovIndex			ΔGovIndex		
	pi	*t*(*p*_*i*_, *ρ*_*i*_)	p-value	pi	*t*(*p*_*i*_, *ρ*_*i*_)	p-value	pi	*t*(*p*_*i*_, *ρ*_*i*_)	p-value	pi	*t*(*p*_*i*_, *ρ*_*i*_)	p-value
1	2	-1.42	0.85	1	-6.07	**	4	-6.43	**	4	-9.48	**
2	4	-4.24	**	4	-5.21	**	3	-3.33	0.06	0	-7.20	**
3	0	-4.72	**	2	-5.46	**	0	-1.30	0.88	0	-6.05	**
4	0	-2.09	0.54	4	-4.57	**	4	-5.05	**	1	-3.63	0.027
5	0	-4.04	**	2	-4.70	**	2	-1.90	0.65	0	-7.52	**
6	0	-2.13	0.52	0	-6.61	**	0	-1.83	0.69	0	-4.39	**
7	0	-3.22	0.08	1	-3.67	0.024	0	-2.22	0.48	0	-6.16	**
8	0	-2.11	0.54	0	-5.37	**	2	1.21	0.99	1	-6.85	**
9	2	-3.31	0.06	0	-4.56	**	0	-2.91	0.16	1	-4.53	**
10	4	-2.09	0.55	1	-4.86	**	0	-2.14	0.52	0	-6.56	**
11	0	-3.17	0.09	0	-4.74	**	0	-2.09	0.55	0	-4.41	**
12	2	-2.30	0.43	4	-4.22	**	0	-2.48	0.34	0	-7.07	**
13	0	-2.52	0.32	1	-3.25	0.074	0	-2.32	0.42	2	-4.20	**
14	1	-2.67	0.25	2	-3.91	0.011	3	-3.99	**	0	-4.66	**
Individual intercepts and trend included in the equation												
$W\bar{t}$ = -3.04, p<0.01				$W\bar{t}$ = -9.76, p<0.01			$W\bar{t}$ = -2.14, p = 0.016			$W\bar{t}$ = -14.74, p<0.01		

Note: ***p<0.001

** p<0.01

*p<0.05.

Source: authors’ results.

Note: ***p<0.001

** p<0.01

*p<0.05.

In terms of the increase in the CPI in the Czech Republic, Poland, and Italy, such trends suggest improvements in the regulatory frameworks to combat corruption, effective efforts of governance to improve financial performance or compliance, upgraded education about corruption. The observed enhancement in anti-corruption measures can be ascribed to a multi-faceted strategy: the strengthening of regulations designed to combat corruption, increased efforts by governance bodies to improve financial accountability and adherence to regulations and intensified educational initiatives to increase public understanding of the consequences of corrupt activities. Collectively, these factors could suggest a deliberate and forward-thinking effort to foster an environment characterized by integrity and openness in administration.

Stability in Germany suggests a consistent anti-corruption policy and an effective legal system that deters corruption or a stable public perception. Germany’s consistent Corruption Perceptions Index (CPI) score suggests the presence of a robust and operative anti-corruption infrastructure. This constancy is representative of a deeply rooted legal framework and uniform policy efforts that are successful in preventing corruption, which in turn mirrors a societal belief in the reliability and honesty of governmental institutions. A stable situation can also be considered the cases of countries like Sweden or Denmark, although the trends are apparently downwards, the minimum level of CPI is over 80, which indicates a low level of corruption in these countries. Decrease in other countries (Spain, Hungary, Portugal, Slovenia) indicate corruption scandals, depreciation of public perception due to media coverage, or changes in the methodology of reporting and detecting corruption. This downward trend could be intensified by media reports or alterations in the methods used to measure and document instances of corruption.

The predominantly negative trends in the Composite Index of Education observed in many countries indicate a reduction in government investment in education, poorly planned educational reforms, or changes in population dynamics that influence the demand for education. On the contrary, the positive trajectory observed in Denmark’s education index indicate a strong societal value placed on education, supported by augmented funding and effective policy changes that align with Danish societal principles.

The Government Index, presenting a spectrum of results, reflects the diverse character of governance practices. High values within the index suggest advancements in government transparency, operational efficiency, and responsibility. Conversely, low values could point to inefficiencies in governmental reforms, changes in political administration, or influences from global political events.

The consistent upward trends in Gross National Income (GNI) per capita observed in the studied countries are encouraging, indicating economic growth, increased productivity, greater foreign direct investment, or improved conditions within domestic markets. These trends are typically indicative of a healthy economy and point to an economic climate that is supportive of further growth.

Thus, the intricate interplay of these indices paints a complex picture of the economic and political landscapes within the Schengen Zone. Policymakers and stakeholders must therefore navigate these dynamics with a nuanced understanding, recognizing that the fight against corruption is inextricably linked with educational initiatives, governance reforms, and economic strategies that are responsive to both national circumstances and global developments.

Overall, the results show heterogeneity between countries and probably mixed stationarity. Therefore, first we employ IPS test, and we analyze the stationarity. First difference transformation may solve the problems related to autocorrelation. Tables 2A-2C describe the results for IPS unit root for each variable and country, as-well as the aggregate decision.

Applying the IPS panel unit root test for heterogenous panel, several interesting results were observed. First, looking at the country specific values, one can observe various results for all the four implied variables. Thus, a mixed combination of stationary and non-stationary time series characterizes the panel data set. Then, looking at the overall $W\bar{t}$ statistics, we assume that GNI is the only series with non-stationary in level.

From an economic viewpoint, a non-stationary Gross National Income (GNI) per capita time series indicate an economic environment that is evolving due to transformative factors like technological advances, shifts in international trade, or demographic changes. Also, non-stationary GNI suggests that a country’s gross national income per capita is influenced by long-term trends or shocks that do not dissipate quickly over time. This indicates a changing economy, possibly undergoing restructuring, which requires adjusted economic policies to manage growth or volatility.

These factors typically lead to structural changes within an economy, which may not be immediately self-regulating, thereby exerting a sustained influence on economic indicators. In contrast, a stationary time series could denote a degree of economic consistency, potentially arising from sound monetary policies, robust regulatory frameworks, and effective governmental actions that have reached a stable state. In this context, a stationary Corruption Perceptions Index (CPI) suggest the effectiveness of anti-corruption strategies or a stable equilibrium in public trust and perceptions of institutional integrity.

The variation in the stationarity of time series across countries highlights the need for economic policies that are not one-size-fits-all but are instead finely tuned to the specific economic realities of each country. Countries with non-stationary GNI series may require policy measures that focus on economic diversification, investment in sustainable industries, or education reforms to build a skilled workforce capable of adapting to changing economic conditions.

The other indicators like CPI, lnCPI, EduIndex and GovIndex have no unit root in level. At the governmental level, the mixed results for the GovIndex suggest that governance quality and effectiveness vary significantly across countries. This is due to differences in political stability, the efficacy of public institutions, or the strength of legal systems. Non-stationary government indices indicate periods of reform or shifts in governance strategies, which have long-lasting impacts on institutional performance and public trust.

Taking into account the heterogeneity of unit root results on the cross-section level, we test the presence of unit root even for the first differences of the variables. In consequence, the homogeneity significantly increases. Considering the obtained results, the following stages are calibrated to estimate the model presented in the method section.

The different variability between countries indicates that economic, political and social factors vary significantly from country to country. This heterogeneity reflects distinct economic contexts that can influence the trends of the time series for the variables in question. Time series that are stationary indicate relative stability in the behavior of the respective economic indicators. For policy makers and analysts, this suggests that, in the absence of external shocks, they can expect relatively constant economic conditions in the future.

Moving to the first difference (where year-on-year changes are analyzed instead of absolute levels) where increased homogeneity was observed, the results suggest that although countries may have different economic trends over time, they behave similarly when faced with year-on-year changes. This observation can be useful for estimating economic models that assume similar responses to external policies or events in different countries.

The results also indicate that individual country differences need to be considered when estimating economic relations at the panel level. From an economic point of view, these results indicate a need for differentiated policies tailored to country-specific conditions, and the importance of understanding the long-term dynamics of economic variables, such as GNI, in economic modelling and forecasting.

First, estimating the parameters of M1 model, we achieve the results from Table 3.

**Table 3:** Main results for FE model regarding the drivers of CPI.

Dependent variable = ln(CPI)	Coefficients ln(CPI)	Dependent variable = Δln(CPI)	Coefficients Δln(CPI)
constant	Various values	Constant	Various values
Δln(GNI)	0.182	Δln(GNI)	0.0552
EduIndex	-0.026**	ΔEduIndex	-0.0251**
GovIndex	-0.066***	ΔGovIndex	-0.0086
R2	0.23	R2	0.038
pDW	0.50***	pDW	1.73*
F-statistic	24.5***	F-statistic	3.315*

Note: ***p<0.001

** p<0.01

*p<0.05.

The econometric results show via Fisher statistic an overall significance (p<0,05) for both models. But looking on the individual significance of the coefficients we may retain a statically significant (p<0,01) impact of Education Index and a significant impact of GovIndex only in the level model. Economic development does not drive the corruption index, regardless of the model used. Going further, the capacity of the model to explain the variation of dependent variable is very low, as R2 is near to 20% for the first case and only 4% in the second try. The Durbin-Watson, DW, underlines the existence of a serial correlation of idiosyncratic errors in both models.

Economic development does not seem to influence the corruption index in any of the models. This suggests that there are other factors driving corruption besides economic development. The unexpected lack of influence from economic development on corruption suggests that corruption is a multifaceted phenomenon not easily captured by traditional economic models This calls for a broader exploration of underlying factors that might be at play. Factors such as political stability, robustness of democratic institutions, and adherence to the rule of law are critical in countering corruption. Corruption driven by political motives can thrive in settings where democratic governance is not firmly established, regardless of a country’s economic status. The presence of political favoritism, entrenched favoritism networks, and a lack of resolve to enforce anti-corruption legislation can all significantly contribute to ongoing corruption.

Additionally, global economic pressures, international business practices, and cross-border transactions can contribute to corruption, especially when transnational corporations operate in environments with disparate levels of governance and regulation. By accounting for individual country differences and considering year-on-year changes, policymakers and analysts can develop more accurate economic models and make informed decisions to address country-specific challenges and promote sustainable economic growth.

From an economic point of view, even if the EduIndex has a statistically significant impact, its negative coefficient indicates that an increase in the value of the EduIndex is associated with a decrease in the Corruption Perception Index (CPI). According to the definition of CPI, decrease of CPI value indicates increase of national corruption level. [50]. This suggests that a higher value of education is associated with an increase in corruption, which corresponds to an increase of corruption in the public perception.

The findings indicating a negative impact of the EduIndex on the Corruption Perception Index (CPI) may initially appear counterintuitive, as education is often seen as a contributing factor to corruption reduction. However, the interpretation of this negative relationship is more complex and may not necessarily suggest a direct causality. Here are some possible interpretations: a. As the level of education rises, citizens’ expectations regarding governance and transparency may also increase. This could lead to a sharper perception of corruption even if the actual level of corruption does not rise. Consequently, the CPI might reflect an increased awareness of corruption, rather than an actual increase in corruption; b. here may be a reverse causality where countries with higher levels of corruption invest more in education to combat the phenomenon, which could explain the negative association observed in the data; c. In some countries, education alone may not be sufficient to combat entrenched cultural practices or systemic issues that allow corruption to thrive. [43,48]. Therefore, an increase in the level of education in these contexts might not necessarily reduce corruption.

This analysis uncovers a nuanced relationship between education and corruption, extending to encompass governance and economic performance. While previous research implies a negative correlation between corruption and educational attainment, our study reveals a deeper connection. Building on Agerberg’s [43] argument that corruption undermines the positive impact of education on governance, our findings present education in a unique light. This suggests a need for innovative educational strategies to enhance individuals’ abilities in addressing corruption effectively. Implementing such approaches within professional education can foster critical thinking skills, particularly in combating corruption, benefiting economic prosperity and governance effectiveness across Schengen member countries.

The significant impact of education on reducing corruption aligns with theories that suggest education fosters critical thinking, civic responsibility, and awareness of rights, which in turn empower citizens to demand accountability. However, the specific mechanisms through which education influences corruption perceptions—whether through improved civic engagement, changes in public values, or increased scrutiny of public officials—require further investigation.

Both atypical economic and statistical contexts drive us to quit the above models and to investigate a dynamic approach as defined in the M2 classes from the data and methods section. In the present context, where the main driver is rather an ambiguous effect and the described models do not pass the econometric validation, the values of the specific cross-sections are rather meaningless impact.

For these reasons, we have resorted to a dynamic approach, presented in Table 4, that uses methods that take into account changes and evolution of the data over time, as opposed to static models that have not provided satisfactory results.

**Table 4:** Results for M2a class of models for CPI modelling.

Dependent variable	ln(CPI)		
Covariates	Model 1	Model 2	Model 3
*Ln*(*CPI*)_*t*−1_	1.14211***	1.146***	1.1295***
*Ln*(*CPI*)_*t*−2_	-0.14118*	-0.1432**	-0.126x
GNI	0.00025	-0.00005198	-0.002023
*GNI*_*t*−1_	-0.00037	0.0000136	0.000373
*GNI*_*t*−2_	-	-0.0003689	-0.00263
*GNI*_*t*−3_	-	-	0.0002855*
EduIndex	-0.01578	0.0041	0.0074
*EduIndex*_*t*−1_	0.004997	0.0121	0.01204
*EduIndex*_*t*−2_	-	-0.0137*	-0.02056x
*EduIndex*_*t*−3_	-	-	0.00913
GovIndex	-0.00689	-0.1247**	-0.01301**
*GovIndex*_*t*−1_	0.00614	-0.008950	-0.01325
*GovIndex*_*t*−2_	-	0.02190***	0.01244*
*GovIndex*_*t*−3_	-	-	0.01441***
Estimation method	Panel GMM	Panel GMM	Panel GMM
W matrix	Iid. errors	Iid. errors	Iid. Errors
Estimation	One-step	One-step	One-step
Sargan-Hansen J-statistic	p-value = 1	p-value = 1	p-value = 1
F-statistic (slopes)	p-value<0.001	p-value<0.001	p-value<0.001
F-statistic (time dummies)	n/a	n/a	n/a

Note: ***p<0.001

** p<0.01

*p<0.05.

The results presented in Table 4 point to an overall significance of the proposed models. Then, due to low risk associated to the coefficient of *Ln*(*CPI*)_*t*−1_ variable, we confirm the existence of an autoregressive relation of corruption index. A positive and supra-unitary value forms the idea that an increase of corruption perception level in present times, will drive a higher level of perception in the future. A plausible economic explanation involves the impact of economic diversification and the informal sector. Economies reliant on a few sectors are more susceptible to corruption due to concentrated economic control, whereas economies with a broader industrial base might spread economic power in ways that discourage corruption. Similarly, expansive informal economies, which function without regulatory scrutiny, foster and sustain corrupt dealings, thereby solidifying adverse public perceptions over time.

Apparently, the Gross National Income per capita indicator does not capture enough information, thus such findings may explain the corruption perceived level. A possible explanation is that the level of economic development does not affect in a significant manner the corruption or maybe other indicators may be more relevant. From an economic perspective, the disparity in wealth distribution within nations explain why higher Gross National Income (GNI) per capita does not necessarily correlate with lower levels of perceived corruption. Significant income inequality may negate the apparent benefits of economic growth, fostering feelings of injustice or marginalization that can lead to corrupt behavior. In contexts where economic benefits are disproportionately allocated, increased GNI per capita values does not correspond with a decline in perceived corruption, especially if a large segment of the population is not experiencing prosperity.

Regarding the EduIndex results, no synchronous association is found, when EduIndex coefficient has a type I error above 0,05 threshold (model 1). But model 2 and 3 from Table 4 suggest that previous levels of EduIndex may influence the CPI. It is generally acknowledged that Education does not transmit its effects instantaneously, but rather with time-lags. However, even if we accept the existence of lag-transmission effects, the negative sign which accompanies the coefficients of *EduIndex*_*t*−2_ in Model 2 and 3 in Table 4, show that increasing support of education links to decrease of CPI Index. These results are in line with the FE model discussed above.

Another significant outcome which can be retained from these models is that lagged GovIndex variables have a statistically significant impact (p<0,05). Moreover, the effect is not transmitted synchronously, neither at lag 1, but rather with 2–3-time lags. Another positive aspect is that coefficients have a positive value. Thus, an increase of Government capacity will lead to an increase of CPI, which implies a reduction of corruption level.

The observed results can be attributed to several potential causes:

- Inertia and resistance within social and economic systems to change: This suggests that the effects of interventions or changes in policies may take time to manifest fully. Social and economic systems often exhibit a degree of inertia, where entrenched practices and structures resist immediate transformation. As a result, the impact of efforts to combat corruption may be delayed, leading to the observed lagged effects.
- Complexity in measuring corruption: Corruption is a multifaceted phenomenon influenced by numerous factors that may not be adequately captured by specific indicators. The intricate nature of corruption makes it challenging to quantify accurately, leading to potential discrepancies between observed outcomes and anticipated effects. Variations in cultural, social, and political contexts further contribute to the complexity of measuring corruption and understanding its impact.
- Role of education in promoting anti-corruption norms and values: Education plays a crucial role in shaping societal attitudes and behaviors towards corruption. However, the effects of education on corruption perception may not be immediate and can take time to materialize. Education fosters norms and values that discourage corrupt practices, but the dissemination and internalization of these values occurs gradually, influencing perceptions and behaviors over time.
- Governmental effectiveness in combating corruption: Administrative and institutional efficiency are fundamental in the fight against corruption. However, the effects of improvements in governmental effectiveness may accumulate gradually, rather than producing immediate results. Strengthening governance structures, enhancing transparency, and implementing anti-corruption measures require time to yield tangible outcomes, contributing to the observed lagged effects in corruption perception.

Table 5 presents the most important results of the M2b class models replacing the M2a class variables with their first differences, searching to improve the homogeneity across countries regarding the stationarity property and to accomplishment of Ahn-Schmidt conditions [70]. Table 5 also captures the dummy-time variables found to be significant at least in one model. The model seems to improve while the number of lags increases, and the coefficient of CPI autoregression relation remains positive but sub-unitary. Thus, using a difference modelling we observe a lower speed of change in the CPI. The other variables included in the model remain significant, but it seems that the GovIndex impact hung its direction. The class 2c model brings more information about CPI change in time. The negative values of 2008, 2009 or 2020 indicate that in the anomalous years, e.g., during the beginning of crises, the perception of corruption seems to be higher than in the less restricted years. The downturns marked by these years are consistent with the idea that periods of economic instability and uncertainty tend to heighten perceptions of corruption. Recessions, characterized by limited resources and intensified competition for economic prospects, often lead to an escalation in corrupt practices, with entities and individuals potentially turning to unlawful activities as a means of financial preservation.

**Table 5:** Results for M2b class of models for CPI modelling.

Dependent variable	Δln(CPI)		
Covariates	Model 1	Model 2	Model 3
*ΔLn*(*CPI*)_*t*−1_	-0.212**	0.210**	0.213**
*ΔLn*(*CPI*)_*t*−2_	-0.0085	-0.062	-0.072
ΔLn(GNI)_t_	0.0248	0.0452	0.063
*ΔLn*(*GNI*)_*t*−1_	0.0093	0.014x	0.130
*ΔLn*(*GNI*)_*t*−2_	-	-0.010	-0.016
*ΔLn*(*GNI*)_*t*−3_	-	-	0.178x
*Δ*EduIndex	-0.0078	-0.0040	-0.0036
*ΔEduIndex*_*t*−1_	0.0093	0.0107*	0.0105*
*ΔEduIndex*_*t*−2_	-0.010	-0.0425	-0.041
*ΔEduIndex*_*t*−3_	-	0.012	0.0121
ΔGovIndex	-0.0125x	-0.015	-0.0110
*ΔGovIndex*_*t*−1_	-0.0262***	-0.027**	-0.0258***
*ΔGovIndex*_*t*−2_	-0.010*	-0.016**	-0.0145***
*ΔGovIndex*_*t*−3_	-	-0.017x	-0.0104x
2004	0.080***	-0.893***	-0.8351***
2005	0.097***	-0.028***	-0.220***
2006	-	-0.219***	0.0811***
2008	-0.0304***	-0.019**	-0.0271***
2009	-0.020*	-	-0.0227**
2015	-0.014x	-0.0191*	0.016x
2020	-0.012**	-	-0.1337x
2022	-0.0240**		-0.0347*
Estimation method	Panel GMM	Panel GMM	Panel GMM
W matrix	Iid. Errors	Iid. Errors	Iid. Errors
Estimation	One-step	One-step	One-step
Sargan-Hansen J-statistic	p-value = 1	p-value = 1	p-value = 1
F-statistic (slopes)	p<0.001	p<0.001	p<0.001
F-statistic (time dummies)	n/a	p<0.001	p<0.001

Note: ***p<0.001

** p<0.01

*p<0.05.

Thus, the negative values observed during these particular years, which coincide with the onset of economic crises, underscore the impact of economic downturns on perceptions of corruption. They highlight the correlation between economic instability and an uptick in perceived corrupt activities, emphasizing the need for heightened vigilance and anti-corruption measures during times of crisis. Addressing corruption during such periods is crucial for safeguarding societal integrity and fostering trust in institutions, thereby promoting sustainable economic recovery and resilience.

In summary, the results of the M2b class models suggest that the perceptions of corruption are not only shaped by long-term economic and governance trends but are also sensitive to short-term economic fluctuations, policy shifts, and crisis events. Understanding these dynamics requires an appreciation of both the immediate economic environment and the broader, more gradual processes of policy implementation and institutional adaptation.

### 4.3. Considerations on the implications of the study

The results of this study provide interesting insights into theoretical, academic, and empirical research. The assessment of corruption is complex, as evidenced by previous studies. Perception theories or behavioral judgements receive consistency by combining theory with contextual or qualitative analysis. The results of this study show that the assessment of corruption by individuals differs over time and in different contexts, even if, apparently, the legislative framework or injunction measures against corruption rely on common regulations, as is the case in the Schengen countries included in the data corpus. People’s perceptions of corruption are an evocative indicator for establishing comparable criteria between countries in terms of the measures taken by governments and their responsible effectiveness in the fight against corruption.

Our proposed approach uses an original combination of driver mechanisms for corruption, highlighting education in a pivotal role. The results indicate that education strengthens individuals and organizations’ capacity to assess and proactively mitigate risks. Education imprints values like ethics and moral compliance on citizens and plays a fundamental role in the long-term fight against corruption. Robust education stimulates people into a most likely proactive attitude to fight corruption and amend fraud conditions. These results are consistent with previous studies that have argued for the importance of education to enhance social well-being and promote economic growth with strong roots in continuous professional development. The strategic importance of education in the financial and judicial dimensions is undeniable, and the results of our study strongly support this statement.

As other researchers pointed out, the fight against corruption entails diverse resources and demands authorities, companies, and citizens to join forces. Several studies pointed to auditing to secure a key role in mitigating or preventing these phenomena especially in the public sector, where auditors are deemed to be the forefront on the common public interest [6,72]. Confidence in governance gains greater consistency when it receives the support or confirmation of effective governance measures through the certification of an independent professional. The present study supports these approaches and contributes to increasing awareness of the practical importance of strengthening professional development measures through new knowledge in the recognition or combat of fraud, both in accounting and in the legal world. As we all know, the effectiveness of anti-corruption capacity and ambitions is possible through the political will to actively implement a favorable legislative framework for sanctioning fraudulent acts. In addition, the results of this paper can contribute to better adapting professional training or programs to provide proficient advice and establish core competences in the fight against corruption.

The findings of this research offer illuminating perspectives into the multifaceted nature of corruption assessment, an endeavor that melds theoretical frameworks with empirical scrutiny. By integrating perception theories and behavioral judgments with a rigorous analysis of context, this study not only corroborates previous academic insights but also carves a novel path in understanding the temporal and contextual variability of corruption perceptions.

### 4.4. Limitation and future research directions

In assessing the complex landscape of corruption perceptions within the Schengen Zone, this study has clarified several important patterns and relationships. However, it is crucial to acknowledge the inherent limitations that accompany our findings, as they provide context for the study’s contributions and directions for future research.

Firstly, the reliance on perception-based indices, such as the Corruption Perception Index (CPI), introduces subjectivity into the analysis. While perceptions are valuable for understanding how corruption is viewed by the public and business, they do not always correlate with actual levels of corruption. Perceptions can be influenced by media coverage, public opinion, and cultural attitudes, which may not accurately reflect the reality on the ground.

Secondly, the study faces the constraints of cross-national comparisons. Each country’s unique historical context, governance structure, and economic environment present challenges in creating perfectly comparable measures of corruption.

The economic focus also means that other potentially influential factors, such as cultural norms, technological advancements, and international relations, may represent future directions for study to enhance the contextual perception of corruption. The interplay between corruption and these broader societal dimensions is an area that requires further exploration.

These limitations serve as a starting point for subsequent research. They highlight the need for continuous refinement of methodologies, expansion of data sources, and incorporation of interdisciplinary perspectives to deepen our understanding of corruption and its many facets. Future research efforts can build on this foundation, exploring the uncharted territories that this study has brought into focus. By outlining the study’s limitations in this integrated manner, we can provide a transparent critique of the research while setting the stage for further inquiry and improvement in the field of corruption studies.

## 5. Conclusions

The results of the study highlighted the sensitivity of perception of corruption to fluctuating economic and political conditions, the importance of stationarity and comparability in cross-national analyses, and the need to consider the lagged effects and the temporal context in assessing the relationship between drivers and corruption. Drawing from existing understandings of corruption perception across various geopolitical regions, this study offers a novel outlook on corruption perception within the Schengen zone. It explores how economic performance, governance, and education intersect to shape perceptions of corruption at different levels of inference.

The evolution slopes of our sample indicators have drawn interesting comparable patterns to indicate public perception of corruption, education, economic performance, and governance in the analyzed Schengen countries. Improvements in the fight against corruption have been noticed in countries like Czech Republic, Poland, and Italy, while stationary patterns have been observed in Germany. The composite index of education (EduIndex) shows that, in most sample countries, education generally appears to have deteriorated. This suggests a reduction in education quality, access, or investments. In terms of the Government Index, mixed positive and negative values pointed to the lack of uniform trends in the sample countries. Some countries seem to have improved government effectiveness or quality, while others were facing decline, revealing different levels of maturity for individual assessment in the Schengen zone.

With regard to the impact of economic crises on the perception of corruption, the negative values for 2008, 2009 and 2020 indicate that in abnormal years, such as the beginning of the crises, corruption perception is deemed higher. This indicates that during economic crises, government systems are perceived as being more vulnerable to corruption due to economic turmoil, uncertainty and possible rapid changes in policies and regulation. The results align with previous research highlighting that periods of economic instability and uncertainty tend to exacerbate perceptions of corruption within societies.

The outcomes of this analysis shed new light on the complex correlation between education and corruption, expanding the framework to encompass governance and economic performance incentives. While prior studies indicate a negative correlation between increased corruption and the educational attainment of local populations, our study unveils a deeper connection. Agerberg (2018) argued that corruption undermines many of the positive impacts of education on governance, a notion further developed by our findings, which cast education in a distinctive perspective. The findings suggest the potential necessity for novel educational approaches to bolster individuals’ cross-sectional competencies in accurately assessing and addressing corruption. This study indicates that communities within the analyzed Schengen member countries would benefit from fostering critical thinking skills within professional education, particularly aimed at addressing corruption. This would cultivate a unified understanding and approach towards corruption, advantageous for economic prosperity and the effective realization of governance objectives for the collective welfare of the community.

It was not possible to establish a strong causal link between economic growth and corruption. The CPI’s autoregression coefficient remained positive and sub-unitary, indicating a slower rate of change in the CPI. In simple words, while corruption tends to persist over time, changes in corruption perceptions are slower than initially assumed. With respect to the perceptions of governance affecting corruption, the study indicated that changes of GovIndex are relevant for the assessment of corruption. Although it was initially believed that increased government capacity led to a reduction in corruption, the results highlighted the complexity of the relationship between governance and perceived corruption, which is sensitive to context, timeframe, institutional background, or political trends.

Corruption usually entails intent to bend the law with illegal and deliberately hidden activities, that are revealed generally through scandals or prosecutions. This makes it very difficult to measure or point directly to their drivers. The fight against corruption is a long-term process that requires proactive collaboration between the authorities, academics and citizens, robust education, and a strong regulatory framework. Eradicating corruption is an ambition that should not only be read in papers but should be transformed into a change in the way people, the authorities and businessmen think and act.

These results encourage the development of innovative anti-corruption policies and promote a societal shift towards integrity and principled governance. The findings of this study emphasize the potential for impactful change, inspiring a movement towards a more ethical and resilient approach to governance and economic challenges.

### Appendix A

This annex presents relevant adjustments for the main aspects of regression models used during the study.

### Appendix B

List of R-Studio packages used in the analysis: foreign, binr, grid, dplyr, tidyverse, dtplyr, openxlsx, WriteXLS, readxl, readr, magrittr, geofacet, zoo, PerformanceAnalytics, ggcorrplot, GGally, plm, pdynmc.
